# Sirt1 coordinates the mitochondrial UPR and myocellular proteostasis to preserve muscle integrity during muscle atrophy in zebrafish

**DOI:** 10.3389/fcell.2026.1761278

**Published:** 2026-03-11

**Authors:** Qing Chen, Yu-Lun Chang, Miao-Wen Hung, Po-Lin Lee, Chen Hsin, Yi-Fan Lin

**Affiliations:** 1 Institute of Biotechnology, College of Life Sciences and Medicine, National Tsing Hua University, Hsinchu, Taiwan; 2 Department of Medical Science, College of Life Sciences and Medicine, National Tsing Hua University, Hsinchu, Taiwan; 3 Department of Life Science, College of Life Sciences and Medicine, National Tsing Hua University, Hsinchu, Taiwan

**Keywords:** mitochondrial dysfunction, mitochondrial homeostasis, muscle atrophy, myocellular proteostasis, proteostasis, SIRT1, UPR^mt^

## Abstract

**Introduction:**

The decline of mitochondrial homeostasis and proteostasis, the two key cell quality control mechanisms, is the hallmark of aging and age-related diseases. One of the most notable examples is the age-related progressive loss of muscle mass, quality, and strength --a condition known as sarcopenia. In atrophic muscle, mitochondrial dysfunction and proteostasis impairment frequently occur together, indicating a potential association between the decline of mitochondrial homeostasis and proteostasis. However, the mechanism by which these two modes of cell quality control are coordinated remains poorly understood.

**Methods:**

We employed dexamethasone-induced muscle atrophy models in both larval and adult zebrafish to investigate the role of cell stress responses in muscle maintenance. Mitochondrial stress was assessed by measuring the mitochondrial unfolded protein response (UPR^mt^) activity using qRT-PCR and reporter analyses. Proteostasis impairment was evaluated by detecting insoluble polyubiquitinated protein aggregates via Western blotting. Muscle integrity was examined histologically in larval and adult tissues. We performed these assays in *sirt1* loss of function conditions (genetic mutation and pharmacological inhibition). Furthermore, to elucidate the mechanism by which Sirt1 regulates proteostasis and muscle preservation, we inhibited the mitochondrial fatty acid oxidation (mFAO) using etomoxir.

**Results:**

Inhibition of Sirt1 markedly exacerbated muscle deterioration and proteostasis impairment under dexamethasone-induced muscle atrophy in zebrafish. Mechanistically, Sirt1 is required for activation of the UPR^mt^, which in turn promotes expression of the mFAO gene *cpt1b*. Pharmacological inhibition of Cpt1 using etomoxir phenocopied the defects in muscle integrity and proteotoxic stress observed following Sirt1 inhibition. Importantly, enhancement of proteostasis via hormetic heat shock partially rescued the etomoxir-induced muscle defects.

**Discussion:**

We have demonstrated that muscle atrophic stress induced by dexamethasone treatment activates the UPR^mt^ in zebrafish. The UPR^mt^ is part of the activity of a cell stress regulator, Sirt1, to promote mitochondrial function and preserve muscle integrity during muscle atrophy. Notably, suppressing the UPR^mt^ via Sirt1 inhibition leads to protein aggregation and the ultimate loss of muscle mass, indicating a link between mitochondrial function and proteostasis. We have further shown that mitochondrial metabolism plays a role in proteostasis regulation, as pharmacological inhibition of the mFAO exacerbates dexamethasone-induced proteotoxicity. Collectively, our findings have uncovered a previously uncharacterized regulatory mechanism linking UPR^mt^ signaling to myocellular proteostasis, and highlight the activity of Sirt1, which coordinates these two key cell quality control mechanisms, in muscle preservation during muscle atrophy.

## Introduction

Progressive loss of skeletal muscle mass and contractile function during aging—or sarcopenia—is among the most prevalent age-related degenerative conditions (Cruz-Jentoft and Sayer, 2019). As skeletal muscle tissue is largely post-mitotic, the maintenance of cell integrity relies heavily on cell quality control mechanisms, such as mitochondrial homeostasis and proteostasis control (Hood et al., 2019; Labbadia and Morimoto, 2015). With advancing age, muscle cells tend to exhibit signs of mitochondrial dysfunction and proteostasis impairment, indicating a parallel decline of these two modes of quality control (Andreasson et al., 2019; Migliavacca et al., 2019; Hunt et al., 2021). Conversely, the beneficial effect of sarcopenia interventions, such as exercise, has been shown to be mediated through enhancing the activity of both quality control mechanisms (Coen et al., 2018). Despite the intimate association, the mechanisms by which mitochondrial homeostasis and proteostasis control operate in a coordinated manner to preserve muscle integrity during muscle atrophy remain unclear.

The most widely recognized link between mitochondrial dysfunction and proteostasis impairment in muscle atrophy is oxidative stress. As oxidative phosphorylation is compromised in aged muscle, the reactive oxygen species (ROS) level would increase, leading to protein damages (Andreasson et al., 2019). Indeed, studies have shown that blocking mitochondrial dynamics, namely, fission and fusion, in skeletal muscle leads to elevated ROS level, proteotoxicity, and muscle loss (Lee et al., 2021; Sebastian et al., 2016). Beyond ROS-mediated damages, it is increasingly clear that mitochondrial dysfunction can affect cytosolic proteostasis in more direct manners. One such mechanism is the toxic buildup of mislocalized mitochondrial proteins in the cytosol due to mitochondrial protein import impairment (Wang and Chen, 2015; Nowicka et al., 2021). Interestingly, this mechanism has recently been implicated in muscle wasting and the underlying proteotoxic stress in mice (Wang et al., 2022). Mitochondria can also engage with cytosolic proteostasis via regulating protein translation, folding, proteosome activity, and autophagy (Baker et al., 2012; Wrobel et al., 2015; Magalhaes-Novais et al., 2022). Moreover, these inter-organelle crosstalk pathways are directed by mitochondrial stress–signaling pathways, such as oxidative stress signaling, estrogen receptor signaling, lipid signaling, and the integrated stress response (Jenkins et al., 2021a). Therefore, in the context of muscle atrophy, it is possible that mitochondrial stress–signaling pathways not only promote mitochondrial homeostasis but also maintain proteostasis via the inter-organelle crosstalk to preserve muscle integrity. However, the full extent of the involvement of mitochondria and the mitochondrial stress–signaling pathways in muscle atrophy still needs to be elucidated.

The mitochondrial unfolded protein response (UPR^mt^) is a conserved signaling pathway activated by mitochondrial stress, such as impaired protein import, across a range of physiological and pathological conditions (Lin et al., 2021; Lin et al., 2016; Pellegrino et al., 2014; Shpilka and Haynes, 2018). Upon activation, the UPR^mt^ regulates the expression of genes involved in mitochondrial biogenesis, energy metabolism, and antioxidant defense, thereby promoting mitochondrial homeostasis. Given its essential role in mitochondrial fitness, UPR^mt^ signaling has long been hypothesized to contribute to the protection of muscle integrity under atrophic conditions. Supporting this notion, recent studies have reported that the decline of the UPR^mt^ activity is a key factor in the pathogenesis of both age-related and disuse-induced muscle atrophy in humans and mice (Migliavacca et al., 2019; Xu et al., 2022). In addition to promoting mitochondrial homeostasis, the UPR^mt^ can also contribute to muscle health by regulating cytosolic proteostasis (Kim et al., 2016; Labbadia et al., 2017; Quiros et al., 2017). Indeed, enhancing the UPR^mt^ activity has been shown to suppress protein aggregation and restore muscle integrity in aged muscle (Romani et al., 2021). Together, these findings indicate that mitochondrial homeostasis plays a critical role in myocellular proteostasis regulation. However, the underlying mechanism of the coordination of the two modes of cell quality control in the context of muscle atrophy remains unclear. To address this gap, we developed a zebrafish model to assess the role of the UPR^mt^ in dexamethasone (DEXA)-induced muscle atrophy. We have found that the UPR^mt^ is part of the activity of Sirt1 to promote mitochondrial function in muscle cell. Specifically, Sirt1 and the UPR^mt^ enhance the mitochondrial fatty acid oxidation, which preserves muscle integrity likely via promoting myocellular proteostasis. Thus, our findings have shed new light on the mechanism of myocellular proteostasis control and offered a novel link between mitochondrial homeostasis and proteostasis regulation during muscle atrophy.

## Materials and methods

### Animal ethics

All animal procedures were approved by the Institutional Animal Care and Use Committee (IACUC) of National Tsing Hua University (Protocol No.: 11212H096) and were conducted in accordance with relevant ethical regulations and the ARRIVE guidelines. Zebrafish (*Danio rerio*) were maintained at 28.5 °C in the National Tsing Hua University zebrafish facility under standard husbandry conditions (Westerfield, 1993).

For anesthesia, larval zebrafish were immersed in 0.2 mg/mL MS-222 (tricaine methanesulfonate; Cyrus Bioscience, 101-886–86-2) dissolved in embryonic medium for at least 20 min at 28 °C prior to mounting and imaging. Euthanasia was performed by immersion in 0.2 mg/mL tricaine solution at 4 °C for a minimum of 30 min, using embryonic medium for larvae or fish water for adult fish, prior to tissue collection for protein or RNA analysis.

For larval experiments, each group consisted of at least 12 individuals for RNA and protein extraction, or at least four individuals for other assays. In adult zebrafish experiments, single individuals were used per group. All experiments were independently repeated thrice to ensure reproducibility. Animals were randomly selected from a cohort sized approximately twice the target sample size and were allocated to the experimental groups without consideration of sex, as both sexes were included indiscriminately.

### Zebrafish strains, dexamethasone-induced muscle atrophy, and small molecule treatment

Wild-type zebrafish used in this study is the AB strain obtained from the Taiwan Zebrafish Core Facility at Academia Sinica (TZCAS). The transgenic zebrafish *Tg(hspd1:d2-gfp)* was generated and described in a previous study (Lin et al., 2021). To knockdown *ubl5* (zgc:66388), zebrafish embryos were microinjected at the 1-cell stage with 1 nL–2 nL of 0.225 mM *ubl5*-targeting morpholino (Table 1). To generate muscle atrophy, larval zebrafish were treated with 120 μM DEXA (Adooq) for 2 days, with the drug solution refreshed once after the first 24 h. For adult zebrafish, 150 μM DEXA was administered for 9 days (Sun et al., 2024), with the drug solution refreshed every 2 days. Adult zebrafish aged 6 months to 1 year were selected for the experiments. During treatment, the fish were maintained under a standard 14-h light cycle and fed once daily.

**TABLE 1:** Nucleotide sequences of antisense morpholinos and qPCR primers used in this study.

Morpholino	Sequence
*ubl5* translation blocker	5’–GTCATTACACACCACCTCGATCATC–3′

qPCR primer	Sequence
*18s*_forward	5′-TCGCTAGTTGGCATCGTTTATG-3′
*18s*_reverse	5′-CGGAGGTTCGAAGACGATCA-3′
*clpp*_forward	5′-CAGAGAGCAACAATAAGCCGA-3′
*clpp*_reverse	5′-CAGGTGGAGATGGGATTTAGGA-3′
*hspa9*_forward	5′-GTGGCTGTGATGGATGGAAA-3′
*hspa9*_reverse	5′-ATTCCTACAAGCCGCTCTCC-3′
*lonp1*_forward	5′-GTACCCGAAGTGTTCACCGAA-3′
*lonp1*_reverse	5′-CCTCCTCAACAGCTCCATCA-3′
*hspd1*_forward	5′-ACTCCAGAGGAAATCGCTCA-3′
*hspd1*_reverse	5′-CATGCCCTCAATGATCTCAA-3′
*sqstm1*_forward	5′-AGCGAAAAGTGCTCGATGTT-3′
*sqstm1*_reverse	5′-GCTCTTTTCGTTGCTTGATG-3′
*fbxo32*_forward	5′-GGCAACATCAACATCTGGGT-3′
*fbxo32*_reverse	5′-GCAACGTCATCCCTGTGTTT-3′
*trim63a*_forward	5′-ATTCTCCCGTGCCAACACAA-3′
*trim63a*_reverse	5′-CGAAGCGACAAGTAGGGCAT-3′
*cpt1b*_forward	5′-TGTCCTATCACGCCTGGATC-3′
*cpt1b*_reverse	5′-CGACCAGACAGCAGCTTTAC-3′
*hsp70.1*_forward	5′-GCAGCCTGACAGGACTTTTT-3′
*hsp70.1*_reverse	5′-CAATGCCAATAGCGATTCCT-3′
*acsf2*_forward	5′-AGTCAGCCAGGATCTTT-3′
*acsf2*_reverse	5′-ATAGAGTGGCTCCTTTGGGC-3′
*acadm*_forward	5′-AGACCCGGGCTGTTAAGAAA-3′
*acadm*_reverse	5′-TGCTAGCAGGACATTTGGGA-3′
*acat1*_forward	5′-CTACACCCTCAGAGCAG-3′
*acat1*_reverse	5′-AGCCTTGAGTTTGGGGACTT-3′

In the nicotinamide experiment, adult zebrafish were maintained in fish water containing 75 μM DEXA and 10 mM nicotinamide (Sigma) for four consecutive days, with daily replacement of the drug and fish water. To block Cpt1 activity, larval zebrafish at 4 dpf were treated with 10 μM etomoxir (ETO) (Selleckchem) with or without DEXA for 18 h–20 h. To suppress autophagy, 5 mM chloroquine (Sigma) was added to the larval zebrafish medium for 1-h incubation.

### Protein fractionation and Western blotting

Detergent-soluble and -insoluble protein lysates were extracted from zebrafish larvae (12 larval fish per sample) and adult lateral skeletal muscle (caudal section; approximately 5 mg per sample) following a protocol based on a published method (Rai et al., 2021a). In brief, larval samples were homogenized using a handheld homogenizer with a PP pestle in 1% Triton X-100/PBS. The resultant clear solution was centrifuged at 21,000 × g for 10 min in 4 °C to obtain the detergent-soluble faction in the supernatant. The pallet containing the detergent-insoluble fraction is resuspended in 90 μL of 7 M urea/1% SDS/PBS and homogenized until the pallet was dissolved. Benzonase (Sigma, 20 U per sample) was added to remove genomic DNA. The resultant solution was centrifuged at 21,000 × g for 10 min in 4 °C to obtain the detergent-insoluble fraction in the supernatant. Adult muscle samples were subjected to sonication (Qsonica Q2000, 2 s pulse × 30 cycles) before undergoing the same fractionation protocol. Protein samples were separated by SDS-PAGE using an 8% polyacrylamide gel. After electrophoresis, proteins were transferred onto a PVDF membrane. The membrane was blocked with 5% nonfat dry milk in PBS containing 0.1% Tween-20 (PBST) for 1 h, before blotting in the primary antibodies (Ubiquitin, Santa Cruz Biotechnology, SC-8017, 1:1,000; beta-Tubulin, Affinity Biosciences, T0034, 1:5,000). ECL chemiluminescence was used for signal detection. For adult samples, quantification was obtained from three independent experiments, with a total of three fish per experimental group. For larval samples, quantification was obtained from three independent experiments, with at least 45 total larval fish in each experimental group.

### RNA extraction and qRT-PCR

RNA was extracted using TRIzol (Invitrogen) from zebrafish larvae (12 larval fish per sample) or adult lateral skeletal muscles (caudal section; approximately 5 mg per sample). Larval samples were homogenized in 500 µL TRIzol with a syringe and a 30G needle until the solution cleared. Adult muscle samples were broken down with a bead mill (TissueLyser LT, Qiagen) in 500 µL TRIzol, before homogenization with a needle. cDNA was synthesized from 1 µg total RNA using the ReverTra Ace kit (PURGO). qRT-PCR were conducted as previously described [42]. The primers are listed in Table 1. Fold change quantification was obtained from three independent experiments with at least 45 total larval fish or at least three adult fish in each experimental group.

### Fluorescent staining and imaging analysis

Whole larval fish samples were fixed in 4% paraformaldehyde at 4 °C for at least 18 h. Following fixation, the samples were washed thrice in PBST (0.2% Tween/PBS). Then, the samples are incubated in 0.1% Alexa488-phalloidin (Cell Signaling) at 4 °C overnight. The samples are subsequently washed thrice with PBST. Larvae were then mounted with either body side attached to the bottom of a glass-bottom Petri dish with 0.3% low-melt agarose. Larval skeletal muscle fiber organization was examined under a stereo fluorescent microscope (Nikon, Ti2U) or imaged under a confocal microscope (Zeiss, LSM 710). Quantification of muscle fiber organization impairment was performed by counting the number of detached muscle fiber stumps in each larval fish. For lysosome imaging, larval zebrafish was incubated in a live cell dye, LysoView Green (ABP Biosciences, 10 µM), for labeling acidic organelles. Stained zebrafish larvae were subsequently mounted and imaged under a confocal microscope. Quantification of lysosomal content was performed by measuring the fluorescent intensity in a rectangle area in the trunk—6-somite long from the anus and half-somite wide from the notochord. All imaging analysis results were obtained from three independent experiments, with at least 20 total larval fish in each experimental group.

### Skeletal muscle tissue section and quantification

Zebrafish muscle tissues were fixed in 4% paraformaldehyde, dehydrated in 30% sucrose, embedded in OCT compound (Leica, FSC22), and cryosectioned at a thickness of 12 μm. Hematoxylin and eosin (H&E) staining was performed to visualize and quantify the skeletal muscle fiber cross-sectional area (CSA). For the CSA analysis, 10 individual muscle fibers in each fish were randomly selected and measured using ImageJ software. Quantification was obtained from three independent experiments with three adult fish in each experimental group.

### Larval zebrafish hormesis preconditioning treatment

At 4 dpf, larval zebrafish were first exposed to 37 °C for 2 h and then transferred to 28 °C for 1 h. Larval zebrafish with or without heat-shock preconditioning were then treated with 120 µM DEXA and 10 μM ETO for 8 h, followed by treatment with 5 mM chloroquine for 1 h. After washing out the chemical inhibitors, larval zebrafish were exposed to 39 °C for 45 min. The larval zebrafish were subsequently fixed, imaged, and scored for the muscle fiber organization analysis, as described above.

## Results

### The UPR^mt^ is induced during muscle atrophy in zebrafish larvae

**FIGURE 1:**
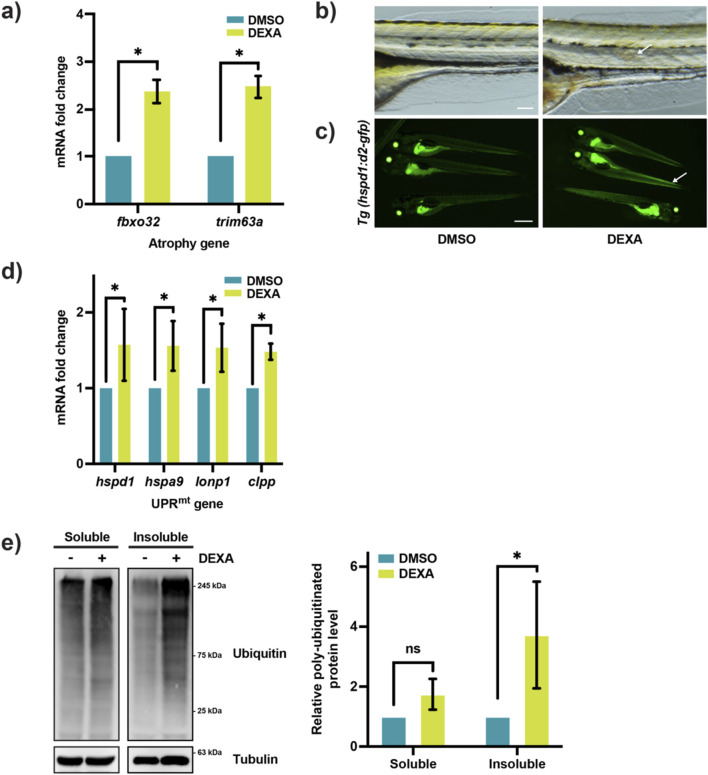
UPR^mt^ is activated during muscle atrophy in zebrafish larvae. **(a)** qRT-PCR analysis of dexamethasone (DEXA)-treated zebrafish larvae, showing the expression of the atrophy genes *fbxo32* and *trim63a* (*p < 0.05, t-test, two-tailed, error bar indicates s.d.). **(b)** Bright-field images of myotome from DEXA-treated zebrafish larvae at 6 dpf. Arrow indicates disorganized muscle fibers. Scale bar represents 0.1 mm. **(c)** Fluorescence images of the UPR^mt^ reporter zebrafish treated with DEXA. Arrows indicate elevated fluorescent signals in the skeletal muscle. Scale bar represents 1 mm. **(d)** qRT-PCR analysis of DEXA-treated zebrafish larvae showing the expression of the UPR^mt^ genes *hspd1*, *hspa9*, *lonp1*, and *clpp* (*p < 0.05, t-test, two-tailed, error bar indicates s.d.). **(e)** Western blot and quantification of poly-ubiquitinated proteins in the detergent-soluble and -insoluble fractions of the protein lysates from DEXA-treated zebrafish larvae (*p < 0.05, t-test, two-tailed, error bar indicates s.d.).

To study the potential involvement of the UPR^mt^ in muscle atrophy, we utilized the UPR^mt^ reporter zebrafish, *Tg(hspd1:d2-gfp*) (Lin et al., 2021). The reporter zebrafish is treated with a high dose of DEXA (100 µM) from 4 dpf–6 dpf for the induction of muscle atrophy. Consistent with what has been shown before (Sun et al., 2024), the treatment results in the upregulation of the two key muscle atrophy genes, *fbxo32* and *trim63a* (Figure 1a). Although the majority of DEXA-treated fish appeared normal in morphology and behavior, some exhibited mild trunk curvature, which indicates defects in skeletal muscle (Figure 1b). Interestingly, the DEXA-induced muscle abnormality was accompanied by activation of the UPR^mt^, as the fluorescent signal in skeletal muscle increased in reporter fish upon treatment (Figure 1c). However, due to the relatively high level of steady-state reporter activity, the UPR^mt^ reporter fluorescence does not provide a robust quantitative measure of the signaling activity. To confirm that the UPR^mt^ is indeed activated by DEXA, we performed qRT-PCR on a panel of the key UPR^mt^ genes, *hspd1*, *hspa9*, *lonp1*, and *clpp*. The result shows that all four UPR^mt^ genes are significantly upregulated following the DEXA treatment (Figure 1d), indicating that the DEXA treatment induces mitochondrial stress in muscle cells. Proteostasis impairment is a hallmark of cell stress phenotype in muscle atrophy. To test whether proteostasis impairment arises in tandem with mitochondrial stress, we measured the level of insoluble protein aggregates, which tend to accumulate in atrophic muscle (Rai et al., 2021b). Specifically, we fractionated protein lysates from larval zebrafish and conducted Western blotting to examine the level of poly-ubiquitinated proteins in the detergent-insoluble fraction. As expected, the results show that DEXA treatment caused a marked increase in high-molecular-weight poly-ubiquitinated proteins in the detergent-insoluble fraction but not in the detergent-soluble fraction, indicating that the DEXA treatment leads to the accumulation of protein aggregates in larval zebrafish (Figure 1e). Our observation demonstrates that mitochondrial stress correlates with proteostasis impairment during muscle atrophy.

### Inhibition of Sirt1 suppresses the UPR^mt^, leading to proteostasis impairment in muscle

**FIGURE 2:**
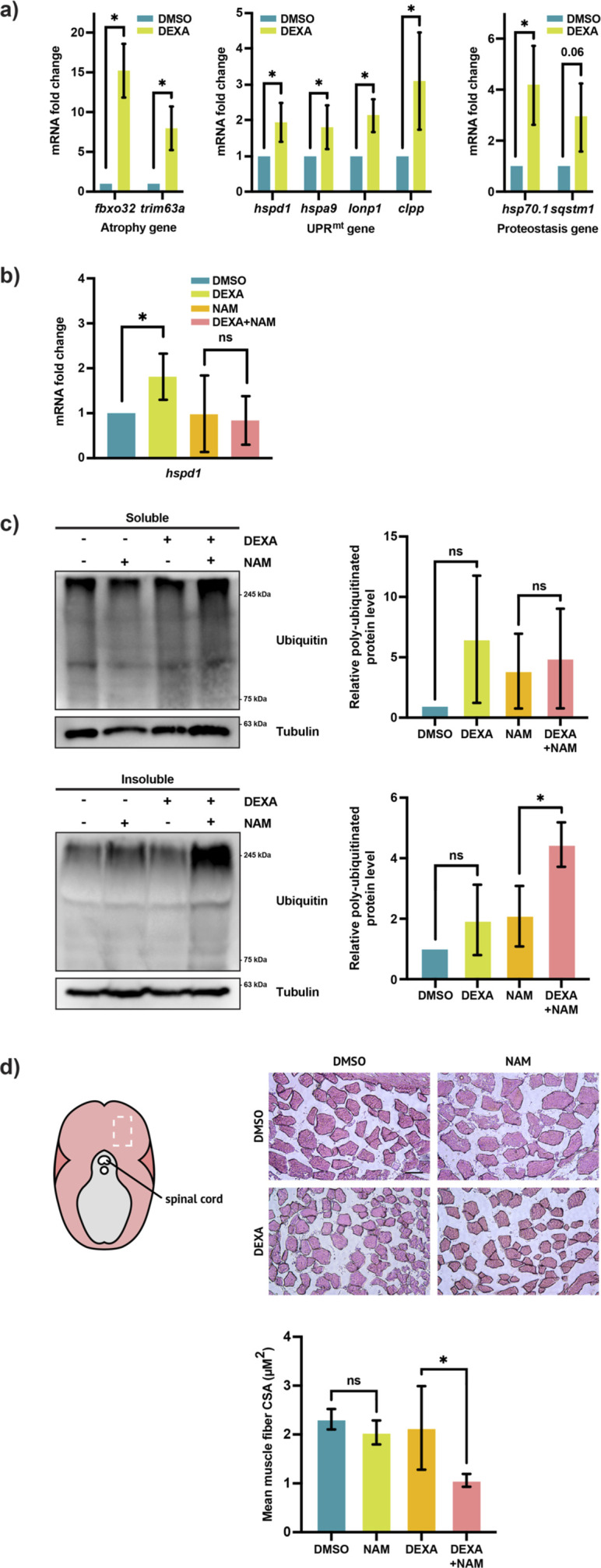
Sirt1 and the UPR^mt^ promote myocellular proteostasis and muscle maintenance. **(a)** Left: qRT-PCR analysis of the skeletal muscle from DEXA-treated adult zebrafish showing the expression of the atrophy genes *fbxo32* and *trim63a* (*p < 0.05, t-test, two-tailed, error bar indicates s.d.). Middle: qRT-PCR analysis of the skeletal muscle from DEXA-treated adult zebrafish showing the expression of the UPR^mt^ genes *hspd1*, *hspa9*, *lonp1*, and *clpp* (*p < 0.05, t-test, two-tailed, error bar indicates s.d.). Right: qRT-PCR analysis of the skeletal muscle from DEXA-treated adult zebrafish showing the expression of the proteostasis genes *hsp70.1* and *sqstm1* (*p < 0.05, t-test, two-tailed, error bar indicates s.d.). **(b)** qRT-PCR analysis of the skeletal muscle from adult zebrafish treated with DEXA and/or nicotinamide (NAM) showing the expression of the UPR^mt^ gene *hspd1* (*p < 0.05, t-test, two-tailed, error bar indicates s.d.). **(c)** Western blot and quantification of poly-ubiquitinated proteins in the detergent-soluble and -insoluble fractions of the protein lysates from the skeletal muscle of adult zebrafish treated with DEXA and/or NAM (*p < 0.05, t-test, two-tailed, error bar indicates s.d.). **(d)** Representative light microscopy images of hematoxylin and eosin (H&E)-stained cross-sections of the skeletal muscle from adult zebrafish treated with DEXA and/or NAM. The images are acquired from the general area of the adult fish musculature indicated by the white rectangle in the diagram to the left. Scale bar represents 100 µm. Histogram showing the average cross-sectional area of the skeletal muscle fibers from adult zebrafish treated with DEXA and/or NAM (*p < 0.05, t-test, two-tailed, error bar indicates s.d.).

To ensure that our observations in larval zebrafish do not reflect a developmental defect and to confirm that the UPR^mt^ is indeed activated in muscle tissue during muscle atrophy, we utilized adult zebrafish (6 months–1 year). Consistent with the observation in larval zebrafish, adult zebrafish treated with DEXA for 9 days exhibit upregulation of the atrophy genes in skeletal muscle tissue (Figure 2a). Moreover, we observed a parallel upregulation of the UPR^mt^ genes and the proteostasis genes, *hsp70.1* and *sqstm1*, confirming that mitochondrial stress correlates with proteostasis impairment in atrophic muscle.

We then sought to determine the role of the UPR^mt^ in muscle atrophy. We have previously shown that UPR^mt^ is activated by Sirt1-induced mitochondrial biogenesis in the context of zebrafish tail-fin regeneration (Lin et al., 2021). To test whether Sirt1 is also required for UPR^mt^ activation during muscle atrophy, we inhibited Sirt1 by incubating DEXA-treated adult zebrafish in nicotinamide (NAM) and evaluated the effect of NAM on UPR^mt^ activation. The concentration used in this study (10 mM) is within the range previously demonstrated to suppress Sirtuin activity *in vivo* in both zebrafish and mice (Lin et al., 2021; Scatozza et al., 2020). We found that the NAM treatment is able to suppress the DEXA-induced *hspd1* upregulation (Figure 2b). Notably, treatment of NAM for the full duration of DEXA incubation, which is 9 days, can cause lethality in zebrafish. To circumvent this, a shorter (4 days) treatment course, which avoids the NAM-associated general toxicity, is adopted for evaluating the effect of Sirt1 inhibition in atrophic muscle. However, in this shorter treatment course, only *hspd1* out of the four UPR^mt^ genes was found consistently upregulated by the DEXA treatment. The involvement of Sirt1 in UPR^mt^ activation led us to examine whether Sirt1 also plays a role in promoting proteostasis. To analyze the effect of inhibiting Sirt1 activity on proteostasis, we fractionated protein lysates from adult zebrafish skeletal muscle and performed Western blotting to examine the level of poly-ubiquitinated protein in the detergent-insoluble fraction. The result shows that the DEXA + NAM treatment significantly increases the accumulation of high-molecular-weight poly-ubiquitinated proteins in the detergent-insoluble fraction (Figure 2c). To evaluate the role of the Sirt1-mediated cell quality control on muscle mass maintenance, we performed skeletal muscle cross-sectioning and H&E staining. The analysis shows that the NAM treatment causes a significant decrease of the muscle fiber cross-sectional area in DEXA-treated zebrafish (Figure 2d), demonstrating that Sirt1 inhibition causes loss of muscle mass during atrophic stress. In summary, the data show that Sirt1 promotes mitochondrial function and proteostasis to protect muscle mass during muscle atrophy.

### *cpt1b* upregulation is the key output of Sirt1/UPR^mt^ activity during muscle atrophy

**FIGURE 3:**
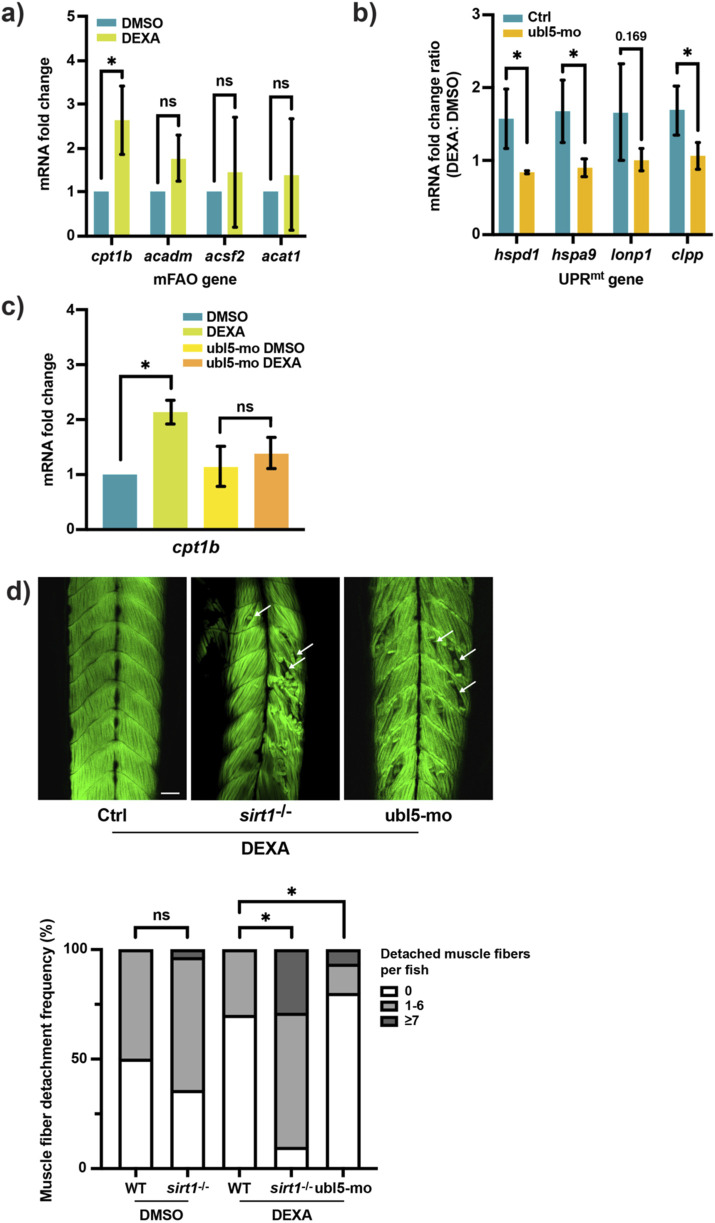
Sirt1 and the UPR^mt^ enhance the mFAO to preserve muscle integrity during muscle atrophy. **(a)** qRT-PCR analysis of the skeletal muscle from DEXA-treated adult zebrafish showing the expression of the mitochondrial fatty acid oxidation (mFAO) genes *cpt1b*, *acadm*, *acsf2*, and *acat1* (*p < 0.05, t-test, two-tailed, error bar indicates s.d.). **(b)** qRT-PCR analysis of wild-type or the *ubl5* knockdown (ubl5-mo) zebrafish larvae treated with DEXA showing the DEXA-induced fold increase of the UPR^mt^ genes *hspd1*, *hspa9*, *lonp1*, and *clpp* (*p < 0.05, t-test, two-tailed, error bar indicates s.d.). **(c)** qRT-PCR analysis of wild-type or the *ubl5* knockdown (ubl5-mo) zebrafish larvae treated with DEXA showing the expression of *cpt1b* (*p < 0.05, t-test, two-tailed, error bar indicates s.d.). **(d)** Representative confocal microscopy images of Alexa488-phalloidin-stained wild-type, the *sirt1* mutant, and the *ubl5* knockdown (ubl5-mo) zebrafish larvae treated with DEXA. Boxplot shows quantification of larvae with 0, 1–6, or ≥ 7 detached muscle fibers (*p < 0.05, Chi-square test. Arrows indicate examples of detached muscle fibers. Scale bar represents 50 µM).

The activation of Sirt1 and the UPR^mt^ indicates that enhancement of mitochondrial metabolic activities is important for muscle preservation during atrophy. To explore this possibility, we performed a targeted transcriptional analysis via qRT-PCR, focusing on the genes involved in lipid metabolism because dysregulation of lipid metabolism is one of the hallmarks of muscle atrophy; moreover, Sirt1 is a key regulator of lipid metabolism in muscle cell (Lai et al., 2024; Gerhart-Hines et al., 2007). We identified carnitine palmitoyltransferase 1b (*cpt1b*), a muscle form of *cpt1*, as a gene upregulated in skeletal muscle in adult zebrafish following the DEXA treatment. Cpt1 is the first component of the mitochondrial fatty acid oxidation pathway (mFAO). It mediates the shuttling of long-chain fatty acids across the mitochondrial outer membrane. Surprisingly, among the several genes in the mFAO pathway, we only found *cpt1b* significantly upregulated by the DEXA treatment (Figure 3a). To test whether *cpt1b* upregulation is dependent on the Sirt1/UPR^mt^-mediated mitochondrial activation, we attempted to examine its expression in the skeletal muscle of DEXA + NAM-treated adult zebrafish. However, we were not able to obtain a conclusive result because of the limitation of the 4-day DEXA treatment, which failed to generate a robust *cpt1b* upregulation. Therefore, we opted to test this idea in larval zebrafish. We inhibited the UPR^mt^ signaling by knocking down one of its transcriptional regulators, *ubl5*, via an antisense morpholino. Confirming its regulatory role in the UPR^mt^ signaling, knocking down *ubl5* dampens the upregulation of the UPR^mt^ genes in DEXA-treated larval zebrafish (Figure 3b). We then tested the effect of *ubl5* knockdown on *cpt1b* expression. Consistent with the result from adult skeletal muscle, *cpt1b* is upregulated in larval zebrafish following the DEXA treatment. However, the *ubl5* morpholino blocks the DEXA-induced *cpt1b* upregulation (Figure 3c). This result suggests that Sirt1 promotes mitochondrial activity via the UPR^mt^ in order to enhance the mFAO during muscle atrophy.

In order to examine the muscle atrophy defect at the cellular level, we next sought to establish a quantitative assay for evaluating muscle fiber integrity in larval zebrafish. To this end, we utilized confocal microscopy to image larval zebrafish muscle fiber organization in the somite myotome based on F-actin (phalloidin) staining. The organization pattern of muscle fibers in the myotome is highly stereotypic and repetitive in the chevron-shaped somite in larval zebrafish. However, despite upregulation of the atrophy genes, the initial imaging analysis did not reveal any apparent muscle fiber abnormalities in the myotomes of DEXA-treated zebrafish. We surmised that this could be because zebrafish larvae tend to be sedentary and, as a consequence, impose insufficient strain on skeletal muscle. To increase muscle strain, we exposed zebrafish briefly to a higher temperature, 39 °C, for 1 h, which is known to stimulate locomotor activity (Gau et al., 2013) at the end of the DEXA treatment course. Imaging analysis shows that the combination of the DEXA and the high temperature treatment indeed causes muscle fiber abnormality, albeit at a low degree of severity. We then utilized this assay to validate the role of Sirt1 in muscle maintenance. Consistent with the reduction of the muscle fiber cross-sectional area in DEXA + NAM-treated adult zebrafish, the larval muscle fiber organization analysis shows that larval zebrafish carrying a *Sirt1* mutation (28 bp insertion in exon1 resulting in a premature stop codon (Lin et al., 2021)) exhibits significantly higher degree of muscle fiber abnormality. Upon close examination, the abnormal muscle fibers appear to detach and recoil from the somite boundaries—the myotendinous junctions—leaving gaps in the myotome (Figure 3d). Notably, the curled muscle fiber remnants indicate that the observed phenotype arises from impaired muscle maintenance rather than impaired development. Previous work has shown that a mutation of *fbxo32* (*atrogin-1*) causes a similar muscle fiber detachment phenotype (Ruparelia et al., 2024a). Furthermore, it has also been shown that muscle fiber detachment is associated with apoptosis. Taken together with our data, these findings indicate that Sirt1 regulates myocellular proteostasis to preserve muscle cell integrity and viability during muscle atrophy. Interestingly, applying the same treatment to the *ubl5* morphant zebrafish also results in a similar increase of the muscle fiber detachment phenotype, compared to that in the wild type (Figure 3d). Therefore, Sirt1 and the UPR^mt^ promote mitochondrial activity, likely the mFAO, to preserve muscle integrity in the context of muscle atrophy.

### Blocking the mFAO activity sensitizes muscle to atrophic stress

**FIGURE 4:**
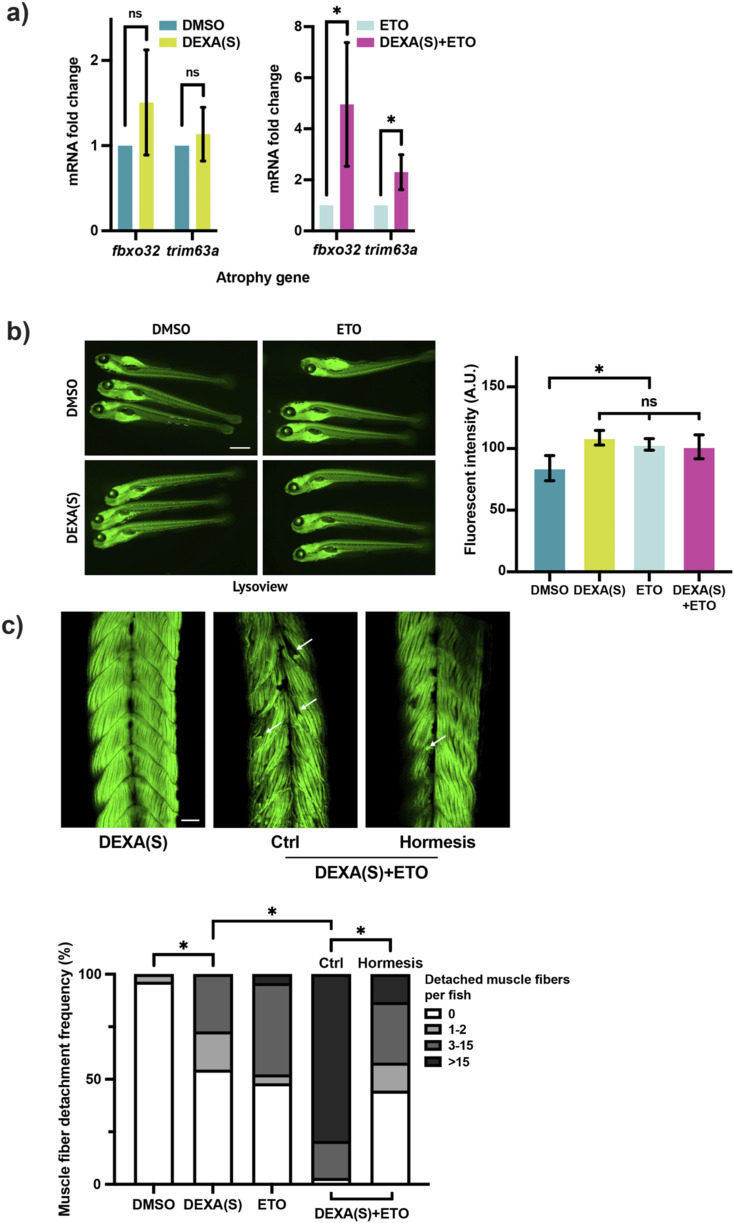
Inhibition of the mFAO sensitizes muscle to atrophic stress. **(a)** qRT-PCR analysis of zebrafish larvae treated with DEXA(S) and/or etomoxir (ETO) showing the expression of the atrophy genes *fbxo32* and *trim63a* (DEXA(S) indicates a short-course DEXA treatment. *p < 0.05, t-test, two-tailed, error bar indicates s.d.). **(b)** Representative fluorescent images of LysoView-stained zebrafish larvae treated with DEXA(S) and/or ETO. Histogram showing quantification of fluorescent intensity (white rectangle indicates the area chosen for the fluorescence quantification. Scale bar represents 1 mm. *p < 0.05, one-way ANOVA and Tukey’s multiple comparison test; error bar indicates s.d.). **(c)** Representative confocal microscopy images of Alexa488-phalloidin-stained zebrafish larvae treated with DEXA(S) and/or ETO with or without the hormesis treatment (arrows indicate examples of detached muscle fibers). Boxplot shows quantification of larvae with 0, 1–2, 3–15, or >15 detached muscle fibers (*p < 0.05, Chi-square test. Scale bar represents 50 µM).

We then went on to test the role of the mFAO in muscle maintenance by blocking its activity with a Cpt1 inhibitor, ETO, in DEXA-treated larval zebrafish. To this end, we designed an interaction experiment in which ETO is administered together with a suboptimal dose of DEXA to test whether mFAO inhibition sensitizes muscle to a milder atrophic stress. Specifically, a shortened (20 h instead of 2 days) DEXA treatment course, hereafter referred to as DEXA(S), was utilized to assess the impact of ETO on muscle integrity. The result shows that the treatment of ETO increases the penetrance of the muscle atrophy defect in DEXA(S)-treated zebrafish, as the treatment of DEXA(S)+ETO leads to a marked upregulation of the atrophy genes, compared to that with DEXA(S) only (Figure 4a). Given the relatively short treatment duration, ETO was used at a concentration of 10 μM, which is higher than concentrations previously reported in zebrafish (Bhan et al., 2023). Although ETO concentrations exceeding 20 µM have been shown to induce nonspecific oxidative stress (O'Connor et al., 2018), oxidative stress measurements using fluorescent imaging with the ROS-sensitive dye H2DCFDA showed that ETO treatment instead led to a decrease in ROS levels irrespective of DEXA treatment (Supplementary Figure S1). Although these results do not completely exclude potential off-target effects of ETO, they indicate that the observed exacerbation of muscle atrophy is unlikely to be driven by increased oxidative stress. We next examined the effect of ETO on muscle fiber organization. To this end, we performed the muscle fiber imaging analysis. However, our initial observation did not reveal muscle fiber abnormality in any of the treatment group. We surmise that this result could be due to the activation of cell protective mechanisms, such as autophagy, that overcome the weaker stress imposed by DEXA(S). To explore this idea, we measured the lysosome activity with fluorescent imaging based on the staining of a pH-sensitive lysosomal dye. Quantification of fluorescent intensity showed that DEXA(S), ETO alone, or DEXA(S)+ETO treatment led to increased lysosomal activity compared with control (Figure 4b), supporting the idea that the activation of autophagy prevents the manifestation of muscle fiber abnormalities. To suppress the protective effect of autophagy, we added chloroquine (CQ) at the end of the DEXA(S) treatment scheme. As expected, the CQ treatment reveals the muscle fiber detachment phenotype. Moreover, we observed a marked increase in detached muscle fibers in the DEXA(S)+ETO treatment compared with DEXA(S) alone (Figure 4c). In summary, these results show that blocking the mFAO sensitizes muscle to atrophic stress.

### The mFAO is involved in myocellular proteostasis regulation

**FIGURE 5:**
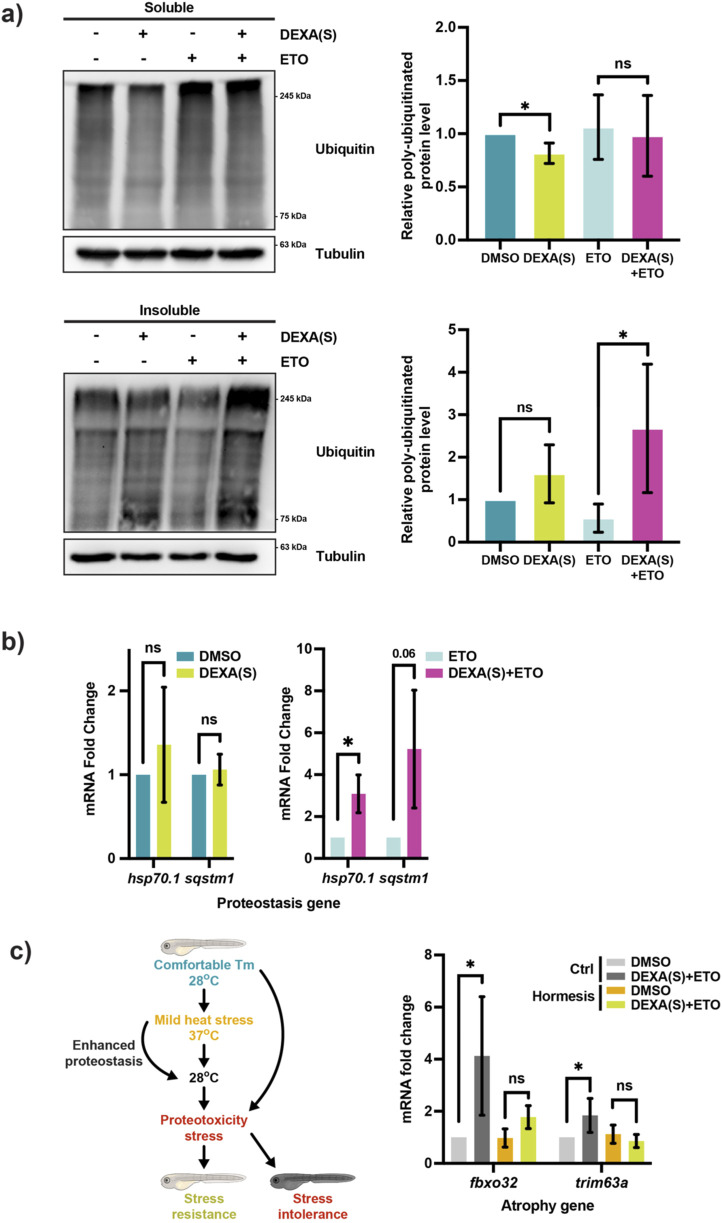
Inhibition of the mFAO exacerbates proteostasis impairment in atrophic zebrafish. **(a)** Western blot and quantification of poly-ubiquitinated proteins in the detergent-soluble and -insoluble fractions of the protein lysates from larval zebrafish treated with DEXA(S) and/or ETO (DEXA(S) indicates a short-course treatment. *p < 0.05, t-test, two-tailed, error bar indicates s.d.). **(b)** qRT-PCR analysis of zebrafish larvae treated with DEXA(S) and/or ETO showing the expression of the proteostasis genes *hsp70.1* and *sqstm1* (*p < 0.05, t-test, two-tailed, error bar indicates s.d.). **(c)** Left: schematic illustration of the hormesis treatment. Right: qPCR analysis of zebrafish treated with DEXA(S)+ETO with or without the hormesis treatment showing the expression of the atrophy genes *fbxo32* and *trim63a* (*p < 0.05, t-test, two-tailed, error bar indicates s.d.).

We then sought to determine the basis of the muscle atrophy phenotype in DEXA(S)+ETO-treated zebrafish. As Sirt1 plays a role in myocellular proteostasis regulation, we wondered whether this effect is meditated via the mFAO. To test this idea, we performed Western blotting to examine the level of poly-ubiquitinated proteins in the detergent-insoluble fraction. Supporting the idea that mFAO is involved in maintaining myocellular proteostasis, DEXA(S)+ETO treatment significantly enhanced the accumulation of high-molecular-weight poly-ubiquitinated proteins in the detergent-insoluble fraction of the protein lysate compared with DEXA(S) alone treatment (Figure 5a). Consistent with this, key proteostasis-related genes, *hsp70.1* and *sqstm1*, were also upregulated in DEXA(S)+ETO-treated zebrafish larvae (Figure 5b). Although direct measurement of protein aggregation or other associated cellular markers would provide a more precise quantification and further characterization of proteostasis impairment, the results presented here demonstrate multiple molecular signatures indicative of increased protein aggregation in muscle atrophy in zebrafish. These results strongly indicate that the muscle atrophy defect caused by mFAO inhibition is due to proteostasis impairment. To validate this idea, we attempted to enhance proteostasis and test whether it can restore muscle integrity in DEXA(S)+ETO-treated zebrafish. To this end, we subjected larval zebrafish to a heat-shock hormesis treatment scheme (Figure 5c). It has been shown in many organisms that a mild heat-shock can trigger a beneficial effect, or a hormetic effect, which is able to mitigate subsequent proteotoxicity-associated conditions, such as neurodegeneration, and can even extend the lifespan in *Caenorhabditis elegans* (Kumsta et al., 2017). We reasoned that if the primary impairment in DEXA(S)+ETO-treated zebrafish is proteostasis impairment, the mild heat-shock-induced hormesis should rescue the muscle atrophy defect. Interestingly, when zebrafish larvae are preconditioned in 37 °C—from 28 °C—for 2 h prior to the treatment of DEXA(S)+ETO, hormesis takes effect and reduces the level of *fbxo32* and *trim63a* expression (Figure 5c). Remarkably, the hormesis effect also rescues the muscle fiber detachment defect in DEXA(S)+ETO-treated zebrafish (Figure 4c). Therefore, enhancing proteostasis is sufficient to ameliorate muscle atrophy-related pathologies, supporting the notion that proteostasis acts downstream of the mFAO activity. Notably, although unlikely, we cannot completely exclude the possibility that a brief heat-shock may influence larval development, which could, in turn, affect muscle integrity independently of proteostasis. Collectively, these findings demonstrate that the enhancement of the mFAO, mediated via Sirt1 and the UPR^mt^, plays a critical role in preserving skeletal muscle integrity by promoting myocellular proteostasis under atrophic stress conditions.

## Discussion

Mitochondrial dysfunction is increasingly being recognized as a contributing factor in the pathogenesis of sarcopenia and other forms of muscle atrophy. While mitochondrial involvement has long been attributed to roles in oxidative stress regulation, our findings provide direct evidence showing that Sirt1 promotes mitochondrial metabolic function—specifically the fatty acid oxidation pathway—via the UPR^mt^, thereby supporting myocellular proteostasis and muscle protection. Therefore, our data reveal a novel molecular link between mitochondrial stress response and cytosolic proteostasis in the context of muscle atrophy. We propose that mitochondria act not only as bioenergetic centers but also as signaling hubs that allow Sirt1 to integrate cellular cues, such as energy status, nutrient availability, and stress signals, to regulate myocellular proteostasis and preserve muscle integrity.

Our work also has demonstrated that zebrafish is a powerful model for identifying novel regulators of muscle atrophy. This model enables the assessment of muscle atrophy at both the cell biological level in larvae and the physiological level in adults. Notably, a small heat-shock protein, Hsbp7, has been shown to be required for proteostasis in the zebrafish heart at both larval and adult stages (Mercer et al., 2018), indicating that the fundamental proteostasis regulatory machinery is conserved across developmental stages. The conservation of key genes involved in muscle maintenance between zebrafish and human provides a strong rationale for modeling muscle atrophy diseases in this system (Daya et al., 2020). Additionally, physiological factors associated with sarcopenia, such as exercise and diet, can be effectively recapitulated in zebrafish (Zou et al., 2022; Sun et al., 2023). The stereotypical organization of muscle fibers in the larval zebrafish myotome offers an additional advantage, enabling quantitative assessment of the effects of genetic or pharmacological interventions on muscle integrity. Notably, a recent study has leveraged this model to analyze a mutation in *fbxo32*, highlighting the potential of using zebrafish in studying muscle atrophy (Ruparelia et al., 2024a).

We have shown that a form of mitochondrial stress signaling, the UPR^mt^, is activated in the skeletal muscle of zebrafish when challenged with DEXA. This result is consistent with the studies in aged muscle, where other mitochondrial stress–response mechanisms, such as fission/fusion, mitophagy, and antioxidation, are essential for muscle mass maintenance (Romanello and Sandri, 2015). Although all of these stress–response mechanisms are important for upholding mitochondrial bioenergetics and ameliorating mitochondrial damages, the involvement of the UPR^mt^ highlights the role of mitochondrial stress-mediated transcriptional reprogramming in preserving muscle integrity, as the UPR^mt^ signaling has been shown to induce the expression of a board range of genes involved in cellular detoxification, metabolism, mitochondrial biogenesis, and the innate immune response (Shpilka and Haynes, 2018). Importantly, ATFS-1, a master regulator of the UPR^mt^ in *C. elegans*, has been shown to be crucial for preventing protein aggregation in muscle during aging, thereby supporting a central role for the UPR^mt^ in muscle maintenance (Romani et al., 2021). Moreover, in human sarcopenia patients, *UBL5* and other UPR^mt^-associated transcriptional regulators have been found to be downregulated (Migliavacca et al., 2019). Together, these findings indicate that the UPR^mt^ plays an evolutionarily conserved role in preserving muscle integrity during muscle atrophy. Here, our data indicate that *ubl5* contributes to the upregulation of *cpt1b* to preserve muscle integrity. Future studies on the mechanism of this regulation, along with other transcriptional outputs of *ubl5*, could greatly facilitate the understanding of the disease mechanism of muscle atrophy.

Diminished NAD^+^—a sirtuin substrate—has been observed in the muscle of sarcopenia patients and is accompanied by downregulation of URP^mt^ signaling (Migliavacca et al., 2019), indicating that the effect of NAD^+^ deficiency can be attributed to the decreased UPR^mt^ activity. On the other hand, replenishing NAD^+^ has been shown to protect muscle against age-related degeneration in a Sirt1- and the UPR^mt^-dependent manner (Romani et al., 2021; Gomes et al., 2013; Zhang et al., 2016). These findings are in line with our observation that Sirt1 preserves muscle integrity via the UPR^mt^ during muscle atrophy. In addition to Sirt1, other sirtuins, such as Sirt3 and Sirt7, have also been shown to play roles in mitochondrial stress signaling in the context of age-related diseases (Mohrin et al., 2015; Jenkins et al., 2021b). However, whether the different sirtuins are involved in age-related muscle atrophy remains to be investigated.

A major risk factor for muscle atrophy is metabolic disorder, and one of the underlying links is myocellular fat metabolism (Kim et al., 2000). We have shown that inhibiting the mitochondrial fatty acid oxidation (mFAO) sensitizes muscle to DEXA-induced atrophic stress. This observation is consistent with the muscle degeneration phenotype found in *cpt1b* knockout mice (Wicks et al., 2015). Moreover, Cpt1b activity has also been found to decline in aged mice, contributing to insulin resistance in muscle (Vieira-Lara et al., 2021). It indicates that Cpt1b is required for protecting muscle integrity against atrophic stress such as aging. Supporting this idea, a recent study in African killifish—a short-lived vertebrate model—has shown that *cpt1b* and *sirt1* are upregulated in concert in certain old individuals, which are able to maintain muscle integrity during aging (Ruparelia et al., 2024b). Further studies are required to directly test the role of the mFAO in adult zebrafish under DEXA treatment or aging conditions to confirm its conserved role in muscle maintenance across different muscle atrophy contexts. Diminished mitochondrial fatty acid oxidation is thought to cause lipid toxicity, leading to insulin resistance and inflammation, in muscle (Li et al., 2022). Here, our data indicate that the mFAO contributes directly to myocellular proteostasis regulation. This finding aligns with emerging evidence showing that lipid metabolism is an important component of the UPR^mt^-mediated cytosolic proteostasis regulation (Kim et al., 2016). Hence, our finding contributes to this growing body of evidence showing that cytosolic proteostasis is regulated via mitochondrial stress signaling, and, more importantly, it demonstrates that this inter-organelle quality control network is critical in the pathogenesis of muscle atrophy. As other novel mechanisms of the mitochondria-mediated cytosolic proteostasis regulation continue to be elucidated, we believe our work demonstrates that the genes involved in this inter-organelle quality control network might be candidate disease genes underlying muscle atrophy.

We propose that in the context of muscle atrophy, Sirt1 and the UPR^mt^ signaling enhance the mFAO to promote myocellular proteostasis and muscle integrity. We speculate that the protective effect of the mFAO is likely mediated via a mitohormesis effect, which is a cytoprotective effect stemming from mitochondrial ROS production and has been shown to enhance proteostasis and promote longevity in *C. elegans* (Labbadia et al., 2017; Schulz et al., 2007). A similar effect has also been reported in healthy aged muscle in mice, where low level of mitochondrial ROS has been shown to preserve mitochondrial function and muscle health (Sebastian et al., 2016). However, it is also possible that the mitochondrial stress–response promotes the mFAO to limit intra-myocellular lipid toxicity, which, in turn, protects proteostasis. Further studies are needed to characterize intra-myocellular lipid metabolism under conditions of Sirt1 or UPR^mt^ loss of function to uncover the mechanisms by which the mitochondrial stress–response regulates myocellular proteostasis. Furthermore, tracking intra-myocellular lipid metabolic changes associated with Sirt1 or UPR^mt^ activity may reveal key lipid metabolites that confer the proteostasis-enhancing effect, as such metabolites have been identified in other contexts (Kim et al., 2016). In summary, our study sheds new light on the critical role of intra-myocellular lipid metabolism in muscle atrophy and may help identify novel therapeutic targets for this condition, which currently lacks effective treatments.

## Data Availability

The original contributions presented in the study are included in the article/Supplementary Material further inquiries can be directed to the corresponding author.

## Supplementary material

SUPPLEMENTARY FIGURE S1 Etomoxir treatment leads to reduced ROS level in zebrafish larvae. Left: representative fluorescent images showing H2DCFDA-stained larval zebrafish treated with DEXA(S) and/or ETO (white rectangle indicates the area chosen for the fluorescence quantification. Scale bar represents 1 mm). Right: histogram showing quantification of fluorescent intensity in the myotome of larval zebrafish. (**p* < 0.05, t-test, two-tailed, error bar indicates s.d.).
