# Supplementary material for: Sirt1 coordinates the mitochondrial UPR and myocellular proteostasis to preserve muscle integrity during muscle atrophy in zebrafish
Source: Front Cell Dev Biol. 2026 Mar 11;14:1761278. doi: 10.3389/fcell.2026.1761278 (PMC13013309; doi:10.3389/fcell.2026.1761278)
Supplement: Supplementary file 1 [file Image1.pdf]

Supplementary Figure S1.

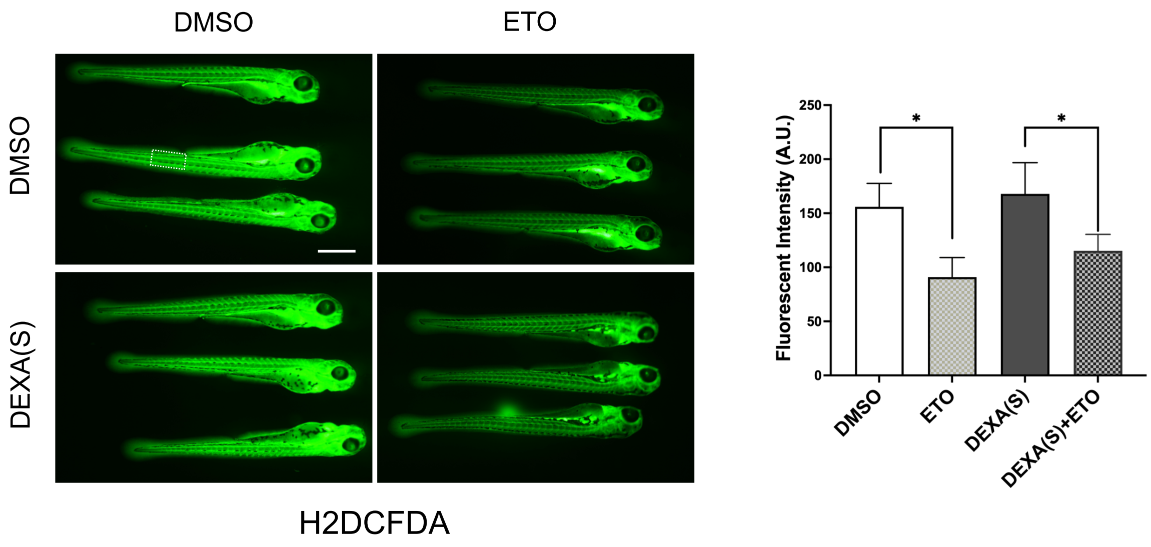

Supplementary Figure S1. Etomoxir treatment leads to reduced ROS level in zebrafish larvae.

Left: Representative fluorescent images showing H2DCFDA stained larval zebrafish treated with DEXA(S) and/or ETO. (White rectangle indicates the area chosen for the fluorescence quantification. Scale bar represents 1mm.) Right: Histogram showing quantification of fluorescent intensity in the myotome of larval zebrafish. (*$p < 0.05$, t-test, two-tailed, error bar indicates s.d.)
